# Cell Treatment for Stroke in Type Two Diabetic Rats Improves Vascular Permeability Measured by MRI

**DOI:** 10.1371/journal.pone.0149147

**Published:** 2016-02-22

**Authors:** Guangliang Ding, Jieli Chen, Michael Chopp, Lian Li, Tao Yan, Qingjiang Li, Chengcheng Cui, Siamak P. N. Davarani, Quan Jiang

**Affiliations:** 1 Department of Neurology, Henry Ford Hospital, 2799 West Grand Boulevard, Detroit, Michigan, 48202, United States of America; 2 Department of Physics, Oakland University, Rochester, Michigan, 48309, United States of America; 3 Department of Neurology, Tianjin Medical University General Hospital, Tianjin, 300052, China; University of Kansas, UNITED STATES

## Abstract

Treatment of stroke with bone marrow stromal cells (BMSC) significantly enhances brain remodeling and improves neurological function in non-diabetic stroke rats. Diabetes is a major risk factor for stroke and induces neurovascular changes which may impact stroke therapy. Thus, it is necessary to test our hypothesis that the treatment of stroke with BMSC has therapeutic efficacy in the most common form of diabetes, type 2 diabetes mellitus (T2DM). T2DM was induced in adult male Wistar rats by administration of a high fat diet in combination with a single intraperitoneal injection (35mg/kg) of streptozotocin. These rats were then subjected to 2h of middle cerebral artery occlusion (MCAo). T2DM rats received BMSC (5x10^6^, *n* = 8) or an equal volume of phosphate-buffered saline (PBS) (*n* = 8) via tail-vein injection at 3 days after MCAo. MRI was performed one day and then weekly for 5 weeks post MCAo for all rats. Compared with vehicle treated control T2DM rats, BMSC treatment of stroke in T2DM rats significantly (p<0.05) decreased blood-brain barrier disruption starting at 1 week post stroke measured using contrast enhanced T_1_-weighted imaging with gadopentetate, and reduced cerebral hemorrhagic spots starting at 3 weeks post stroke measured using susceptibility weighted imaging, although BMSC treatment did not reduce the ischemic lesion volumes as demarcated by T_2_ maps. These MRI measurements were consistent with histological data. Thus, BMSC treatment of stroke in T2DM rats initiated at 3 days after stroke significantly reduced ischemic vascular damage, although BMSC treatment did not change infarction volume in T2DM rats, measured by MRI.

## Introduction

In the clinic, the vast majority (90–95%) of diabetic patients have type 2 diabetes mellitus (T2DM) [1]. Diabetes with concomitant hyperglycemia is a chronic vascular disease [1]. Hyperglycemia induces a variety of biochemical changes within endothelial cells, including those in the cerebral vasculature [2], which instigates a cascade of events leading to vascular endothelial cell dysfunction, and increased vascular permeability in various vascular beds in humans and animal models [3]. Multiple cell and molecular pathways are involved in the diabetes-related changes in the blood-brain barrier (BBB) [4].

Diabetes increases risk of ischemic stroke, stroke recurrence and long-term mortality from stroke, and worsens the overall neurological outcomes after stroke [5, 6]. Abnormalities in glucose metabolism and vascular hemodynamics may play important roles in the pathogenic progress of stroke in diabetic patients [5]. BBB damage and exacerbated secondary hemorrhagic transformation (HT) are consistent consequences of ischemic stroke in diabetic murine animals [7–9]. BBB disruption persists much longer time after stroke in T2DM rats than in non diabetic rats [9].

In non-diabetic rats, treatment of stroke with bone marrow stromal cells (BMSC) significantly reduces vascular permeability in the ischemic brain and improves neurological function within two weeks after stroke, compared with vehicle-treated non-diabetic stroke rats [10]. Surprisingly, BMSC therapy administered at 24h after stroke in rats with type 1 diabetes mellitus (T1DM) fails to improve neurological function, adversely increases BBB leakage and intracranial hemorrhage two weeks after stroke, compared with non-treated T1DM stroke rats [11]. Further investigations are therefore warranted on the effects of BMSC treatment of stroke in T2DM subjects.

Most preclinical studies focus on the measurement of BBB disruption and cerebral vascular permeability during the early stage after stroke in diabetic animals, or employ histological methods [6–8], which do not allow dynamic evaluation and application to patients. In the present study, by employing magnetic resonance imaging (MRI), the temporal characteristics of BBB disruption were monitored weekly up to 5 weeks after stroke in the T2DM rats with or without BMSC treatments. Our primary and secondary endpoints are BBB leakage volume and hemorrhagic volume, respectively, and we prospectively set the endpoints based on our preliminary data. Thus, we can test our hypothesis that these MRI results will clarify, compared with the controls, whether delayed treatment of stroke in T2DM rats with BMSC reduces BBB damage and cerebral hemorrhage, as in non-diabetic rats, or fails to improve and even worsens BBB damage, as in T1DM rats [11]. The present study also provides more detailed information on longitudinal cerebrovascular damage after stroke and therapeutic efficacy of BMSC treatment in T2DM rats.

## Materials and Methods

All experimental procedures were conducted and performed in accordance with the National Institutes of Health (NIH) Guide for the Care and Use of Laboratory Animals and under a protocol approved by the Institutional Animal Care and Use Committee of Henry Ford Health System.

### Animal model and experimental protocol

T2DM was induced in adult (175-200g, 2–3 months) male Wistar rats (Charles River, Wilmington, MA) by feeding them a high fat diet (HFD, 40% of calories as fat) for 2 weeks, then intraperitoneally injecting a single low dose (35mg/kg) of streptozotocin (STZ, Zanosar^®^, Sigma Chemical Co., St. Louis, MO), a naturally occurring chemical that is particularly toxic to the insulin-producing beta cells of the pancreas in mammals, and the HFD was continued for another two weeks. Distinguished from intraperitoneally injecting a single high dose (60mg/kg) of STZ alone for type 1 diabetes, this fat-fed and STZ combination rat provides an animal model for type 2 diabetes, and simulates the human clinic syndrome [12]. Although the HFD/STZ rat is widely employed as a model of T2DM [13–15], it is important to monitor the glucose and insulin levels when using this model, since many factors may impact the T2DM phenotype, such as STZ dose, HFD duration, age and strain of rat [16]. Blood glucose level was measured using test strips for glucose (Polymer Technology System, Indianapolis, IN) for confirmation of hyperglycemia. Rats with 8 hours fasting plasma glucose of ≥300mg/dl were considered diabetic and were selected for middle cerebral artery (MCA) occlusion [17]. Although the rat model of diabetes suggests insulin resistance, we cannot exclude the possibility that this model may be a mixed model of type 1 and type 2 diabetes, since we did not monitor the insulin levels of diabetic rats in this study.[12]

The right MCA was occluded for 2 hours using the filament model [18]. Briefly, a 4–0 monofilament nylon suture, its tip rounded by heating, was introduced into the internal carotid artery lumen through the stump of the external carotid artery and gently advanced into the internal carotid artery 19-21mm past the common carotid artery bifurcation to block the origin of the middle cerebral artery. Reperfusion was initiated by removal of the thread and tying off the distal external carotid artery. During the surgery, rats were anesthetized with a gas flow (1.0L/min) of isoflurane (1.5%) in a 2:1 mixture of N_2_O and O_2_. Buprenex (0.5mg/kg) was intramuscularly injected once post surgery for pain relief. The animals were housed one rat per cage with an enriched environment of small toys in each cage. Room temperature and humidity were controlled at 70°F and 50%, respectively. Rats were observed once per day to make sure that they were not in pain or discomfort, and they were able to function normally and had free to access food and water.

Rats were randomly treated with either BMSC at a dose of 5 million (5x10^6^) cells or an equal volume of phosphate-buffered saline (PBS) as the vehicle via tail vein injection at 3 days after MCA occlusion-reperfusion, the time of stroke and treatments were performed at a fixed time of day. The dose selection is based on a previous study, in which treatment of stroke with human umbilical cord blood cells at a dose of 5x10^6^ initiated 3 days after stroke significantly increased vascular and white matter remodeling in T2DM rats [17]. To avoid neuroprotective effects, the treatment time point of 3 days after stroke in T2DM rats was used only to investigate BMSC-induced neurorestorative effects, as well as to accommodate a wide treatment window. The treated (BMSC) and control (PBS) groups of T2DM rats with stroke were age and body weight matched. Neurological function was monitored by modified neurological severity scores (mNSS) [18] for all animals on days 1, 7, 14, 21, 28, and 35 post stroke.

MRI was performed prior to the MCA occlusion, as an internal control, and at one day and then weekly for 5 weeks after ischemia-reperfusion for all rats. Among a total of 23 rats, seven rats (3 from control and 4 from treated rats) died before completion of 5 weeks of MRI, and were excluded from the study. No autopsy was performed on these rats. Animals did not receive additional medical treatment, but were administered saline twice a day when the animals were dehydrated. After completing final MRI scans, all animals (*n* = 8 for BMSC and *n* = 8 for PBS, respectively) were euthanized at 5 weeks post stroke.

### MRI measurements

MRI measurements were performed with a 7T system (Bruker-Biospin, Ettlingen, Germany). A birdcage type coil was used as the transmitter and a quardrature half-volume coil as the receiver. Pulse sequences included T2-weighted imaging (T2WI), susceptibility weighted imaging (SWI), contrast enhanced T1-weighted imaging (CE-T1WI) with gadopentetate, gadolinium-diethylenetriamine penta-acetic acid (Magnevist^®^, Berlex Inc, Montville, NJ), as the image contrast agent, and dynamic contrast-enhanced MRI (DCE-MRI) with the same single administration of gadopentetate in CE-T1WI.

A fast gradient echo imaging sequence was used for reproducible positioning of the animal in the magnet at each MRI session. During MRI measurements, anesthesia was maintained using medical air (1.0L/min) with isoflurane (1.0–1.5%). Stereotactic ear bars were used to minimize movement, and rectal temperature was maintained at 37±1.0°C using a feedback controlled water bath (YSI Inc, Yellow Springs, OH).

T2WI was acquired using a multislice (13 slices) and multiecho (6 echoes) sequence, with time of echo (TE) as 15ms, and equally to 90ms, time of repetition (TR) as 4.5sec, a 32x32mm2 field-of-view (FOV), 1mm slice thickness and 128x64 matrix. SWI employed a specialized 3-dimensional gradient echo sequence with TE = 10ms, TR = 40ms, flip angle of 15°, 32x32x24mm3 FOV, 256x192x64 matrix, and flow compensation in all three directions. CE-T1WI was composed of two T1-weighted imaging (T1WI) acquisitions, before and 6 minutes after injection of gadopentetate into a tail vein at a dose of 0.4mL/kg. T1WI was acquired using a conventional multislice single spin-echo sequence with TE of 8ms, TR of 500ms and the same other parameters as in T2WI.

The DCE-MRI was performed between the two T1WI acquisitions with a dual gradient echo (DGE) sequence, and allowed to be run 15 seconds as a baseline followed by the gadopentetate injection via tail vein. The TEs of the DGE sequence were 1.5ms and 8ms, respectively; TR was 33ms; 3 continuous slices with 2mm thickness; 32x32mm2 FOV; 128x64 matrix. The scan time was approximately 5 minutes by repeating the DGE sequence 150 times. The time-dependent changes in R_1_ (1/T_1_) were used to form K_i_ map using the following equation:

$$C_{tis}(t)=K_{i}\overset{t}{\underset{0}{\int }}C_{pa}(\tau )d\tau +V_{p}C_{pa}(t)$$ (1)

where K_i_ is the blood-to-brain transfer constant, C_tis_(t) is the tissue concentration of the contrast agent as a function of time, C_pa_(t) is the plasma concentration of the contrast agent as a function of time. V_p_ includes the entire volume of the rapidly filling sub-compartments. Estimates of C_tis_(t) and C_pa_(t) are done via changes in the R_1_ values of cerebral tissue and sagittal sinus blood. Pre-contrast T_1_ was estimated using a set of variable flip-angle spoiled gradient recalled echo images aquired with TR 30ms, TE 4.0ms, flip-angles of 2°, 5°, 10°, 15°, 20° and 25° [19]; where the geometrical parameters were set to precisely match the DGE images after image post-processing.

In this study, we focused on the volume of BBB disruption after stroke in diabetic rats. Thus, CE-T1WI was employed to quantitatively measure the volume of BBB leakage, since the CE-T1WI provides the similar and correlated results with the DCE-MRI,[20] as well as consistent and comparable results with previous measurements in the stroke model of diabetic rat without BMSC treatment.[21]

### Histological staining

Rats were euthanized with ketamine (44mg/kg, intraperitoneal) and xylazine (13mg/kg, intraperitoneal) with the completion of MRI scans at 5 weeks after stroke. Brains were isolated, post-fixed in 4% paraformaldehyde for 2 days at room temperature, and then processed for paraffin sectioning. Coronal sections (6μm thick) were cut from each block and stained with hematoxylin and eosin (H&E) for the evaluation of ischemic lesion and blood red cells in cerebral parenchymal tissue using light microscopy. A stain of fluorescein isothiocyanate dextran (Sigma-Aldrich, St. Louis, MO) conjugated antibody against rat albumin was used for the specific and sensitive determination of BBB leakage.

### Data and statistical analysis

Experiment and data analysis were performed in a blind fashion. MRI image analysis was generally performed with homemade software, Eigentool [9]. T2 maps were obtained pixel-by-pixel from multiple echoes of T2WI by using a linear least-squares fit to the plot of the natural logarithm. SWI was produced using SPIN software [22], and then Eigentool was employed for quantitative analysis after format transformation. Images of CE-T1WI were produced by subtracting the images of pre gadopentetate T1WI from the images of post gadopentetate T1WI with Eigentool. Each first echo image from the DGE sequence was employed to calculate the changes of longitudinal relaxation time constant, T_1_, of the cerebral tissue voxel-by-voxel, and hence to estimate concentration of gadopentetate infiltrated into brain tissue, leading to an estimation of blood-to-brain permeability [23].

Ischemic lesion volumes of MRI were measured on the T2 maps. The mean plus two times standard deviation of the contralateral measurements on the T2 maps was used as a threshold to identify lesion volume. To eliminate the influence of brain atrophy after stroke, the ischemic lesion volume were calculated and expressed as the ratio of lesion volume to contralateral hemispheric volume. The same measurement was performed on the CE-T1WI images [20]. Due to hypointensity of hemorrhage on the SWI intensity image, the mean minus two times standard deviation of the contralateral measurements was used as threshold to identify hemorrhage after stroke [24]. A hemorrhagic spot was counted if more than 3 pixels with 4-connected neighborhoods were demarcated in each axial slice of SWI, and total number and volume of hemorrhagic spots were summarized slice by slice. T1WI and SWI measurements were presented as the direct volumes.

The MicroComputer Imaging Device system (Imaging Research Inc, Ontario, Canada) was used for histological measurements. H&E and albumin stained sections were evaluated at 40x magnifications, respectively. The reactive areas inside striatum were measured (percentage to FOV) under a 40x objective of the optical microscope, using an average of four locations as the histological result.

MRI measurements are summarized as mean and standard deviation. Differences in the MRI data between groups were analyzed by a mixed model of analysis of variance and covariance, respectively. For the longitudinal MRI measurements, the analysis started testing the group and time (without baseline time point) interaction, followed by testing the group difference at each time point (*t*-test) if the interaction or overall group effect was detected at the 0.05 level. Student`s *t*-test was performed for mNSS between the two groups of animals obtained at 5 weeks after stroke.

## Results

**Fig 1:**
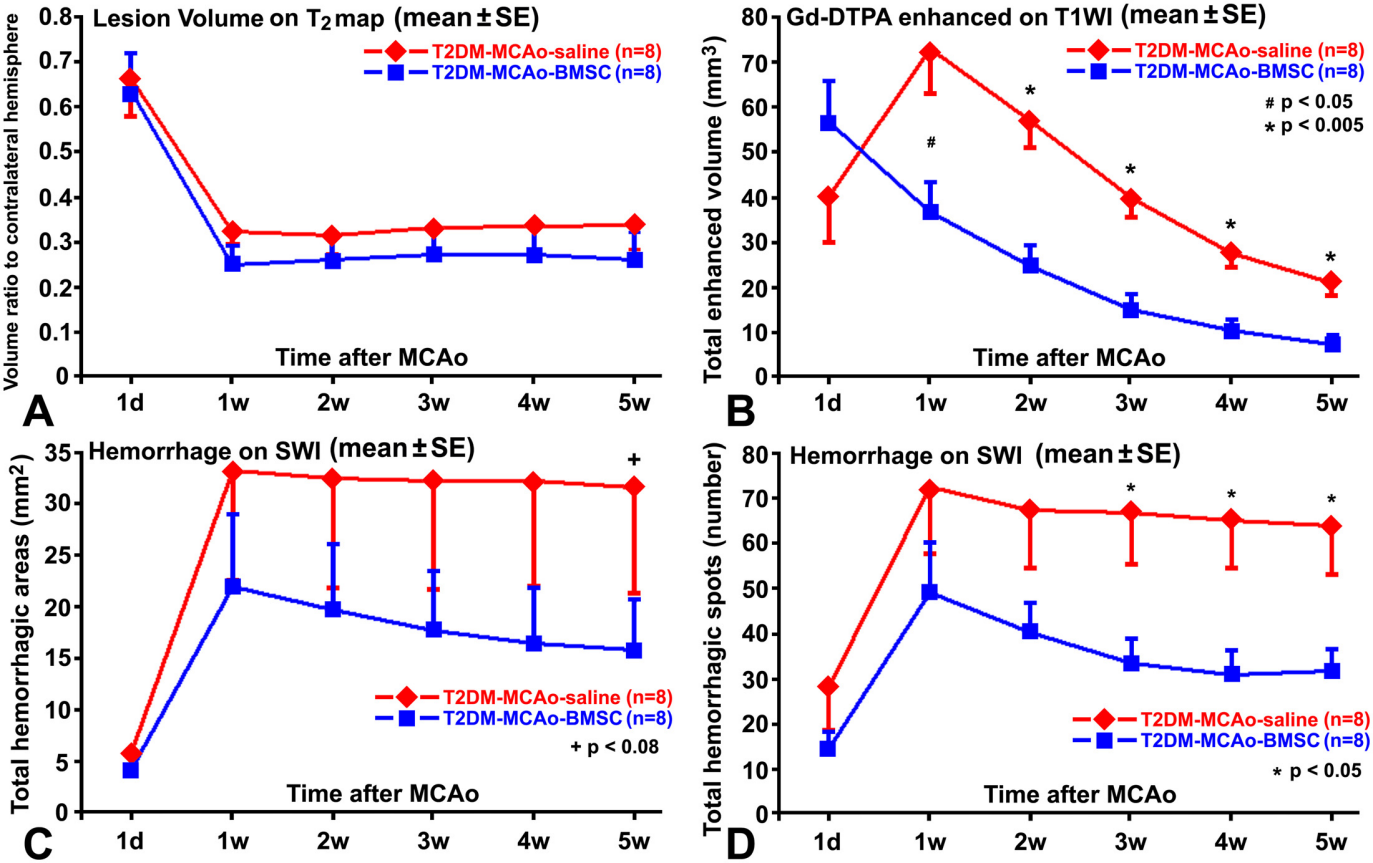
Quantitative measurements of MRI. Temporal traces of the ischemic lesion volumes demarcated from T_2_ maps (A) were close and no significant differences (p>0.05) were detected after stroke, between the BMSC and saline treated T2DM rats. BBB disruption volumes of CE-T1WI with gadopentetate enhancement (B) were significantly smaller in the BMSC treated T2DM rats than in the control rats from 1w after stroke. SWI measurements demonstrated that BMSC treatment marginally reduced hemorrhagic volumes (C) at 5w, but significantly decreased hemorrhagic spots (D) starting 3w, after stroke in T2DM rats.

The 8 hour fasting plasma glucose levels of selected rats measured prior to the induction of middle cerebral artery occlusion was 377.4±50.4mg/dl for the saline treated rats and 385.1±65.4mg/dl for the BMSC treated rats, respectively. Volumes of the ischemic lesion demarcated from T2 maps did not exhibit any significant differences (p>0.1), as indicated in Fig 1A, within 5 weeks after stroke between the BMSC and PBS treated T2DM rats. After the onset of ischemia, lesion volumes experienced a sharp decline from 1d to 1w in all rats (Fig 1A), since brain edema, which caused swelling of the ipsilateral brain hemisphere to approximately 16% greater (p<0.0001) than the contralateral hemisphere at 1d after stroke, quickly faded in the first week. For both control and BMSC treated T2DM rats, stroke lesion volumes demarcated from T2 maps exhibited similar temporal traces (Fig 1A), and were 33.8±15.3 percent (of the contralateral hemisphere) versus 26.1±17.7 percent measured from T_2_ maps acquired at 5w after stroke, or 39.3±12.9 percent versus 38.6±8.9 percent measured from the histological H&E coronal sections using the MicroComputer Imaging Device system, respectively. No significant differences in lesion volume were detected between the control and BMSC treated T2DM rats at 5w after stroke (p>0.05).

**Fig 2:**
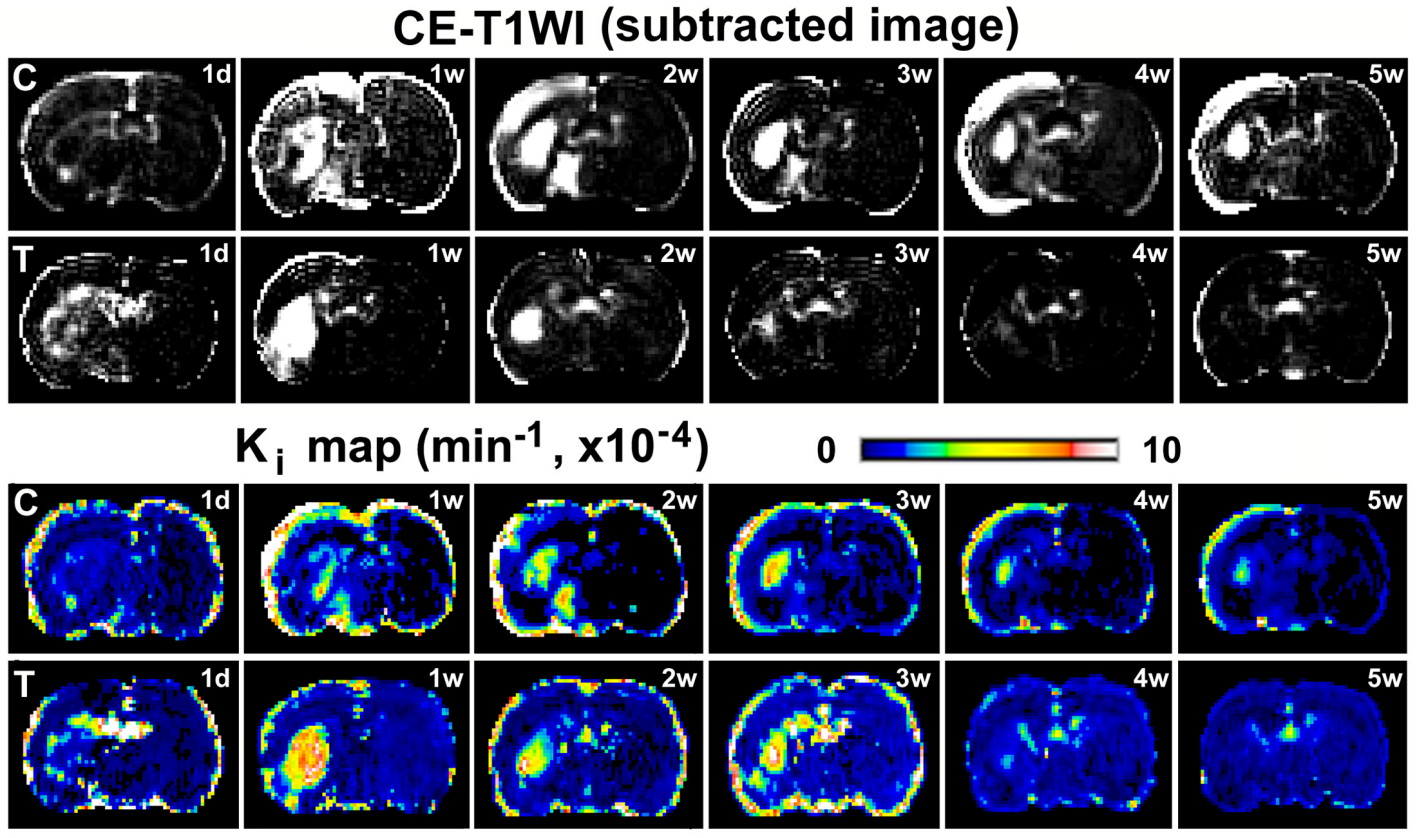
Evolution of BBB disruption by MRI. BBB disruption, with enhancement in the subtracted images of pre and post gadopentetate of CE-T1WI, persisted from 1w to 5w post stroke in saline treated, i.e., control, T2DM rat (C, the top row). In contrast, the hyperintensity regions faded from 4w after stroke in BMSC treated T2DM rat (T, the second row). Permeability K_i_ maps demonstrated the similar results as CE-T1WI did for both control (C, the third row) and the BMSC treated (T, the bottom row) rats.

BBB leakage of gadopentetate persisted for 5 weeks after stroke in the control T2DM rat (upper row of CE-T1WI in Fig 2). Animals, having similar location and volume of stroke lesion, were selected from control and treated groups, respectively, to avoid the effect of ischemic severity on BBB damage, and presented in Fig 2. However, for the BMSC treated T2DM rat, hyperintensity, excluding the ventricle, in the ischemic brain tissue was present in the contrast enhanced images from 1 day to 3 weeks, rapidly faded at 4 weeks, and was hardly detectable at 5 weeks after stroke (lower row of CE-T1WI in Fig 2). The permeability rate maps, derived from DCE-MRI and parameterized by the forward volume transfer constant K_i_ for the estimated transfer rates of contrast agent (i.e., gadopentetate) across the microvasculature into brain parenchyma, showed consistent results as the CE-T1WI for the two representative T2DM rats with or without BMSC treatments (see K_i_ maps in Fig 2).

The quantitative volumes of gadopentetate enhancement with CE-T1WI demonstrated that the BBB damage reached a peak at 1w after stroke in the control T2DM rats, as indicated in Fig 1B. However, by mostly suppressing the increase of BBB damage at 1w after stroke (Fig 1B), BMSC treatment significantly (p<0.05) reduced gadopentetate enhanced volumes from 1w to 5w after stroke in T2DM rats, compared with the control rats.

**Fig 3:**
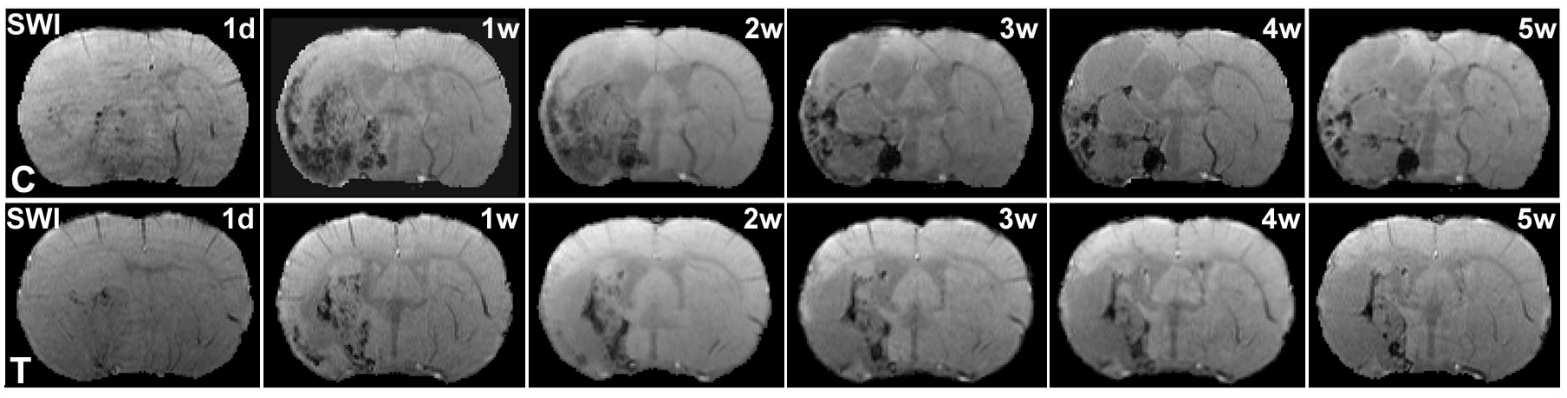
Evolution of hemorrhage by SWI. The evolution of hemorrhage after ischemia were demonstrated in SWI images for the representative control (C, upper row) and BSMC treated (T, lower row) T2DM rats, respectively. Hemorrhagic volumes and spots were larger and more in the control T2DM rat than in the BMSC treated T2DM rat in 5w after stroke.

BBB disruption after ischemia may develop HT and lead to hemorrhage. In the SWI images, the areas of hypointensity, excluding veins, are generally associated with hemorrhage [25]. Fig 3 shows the longitudinal SWI images from the two representative T2DM rats with and without BMSC treatments. The SWI images exhibited much more severe hemorrhage in the control T2DM rat than in the BMSC treated T2DM rat during 1d to 5w after stroke. Quantitative SWI measurements for hemorrhagic volumes, as shown in Fig 1C, demonstrated that the BMSC treated T2DM rats only exhibited marginally significant (p<0.08) smaller hemorrhagic volumes than the control T2DM rats at 5w after stroke. However, hemorrhagic spots, demarcated by SWI, were significantly (p<0.05) reduced from 3w to 5w after stroke in the BMSC treated T2DM rats, in contrast to the control T2DM rats (Fig 1D).

**Fig 4:**
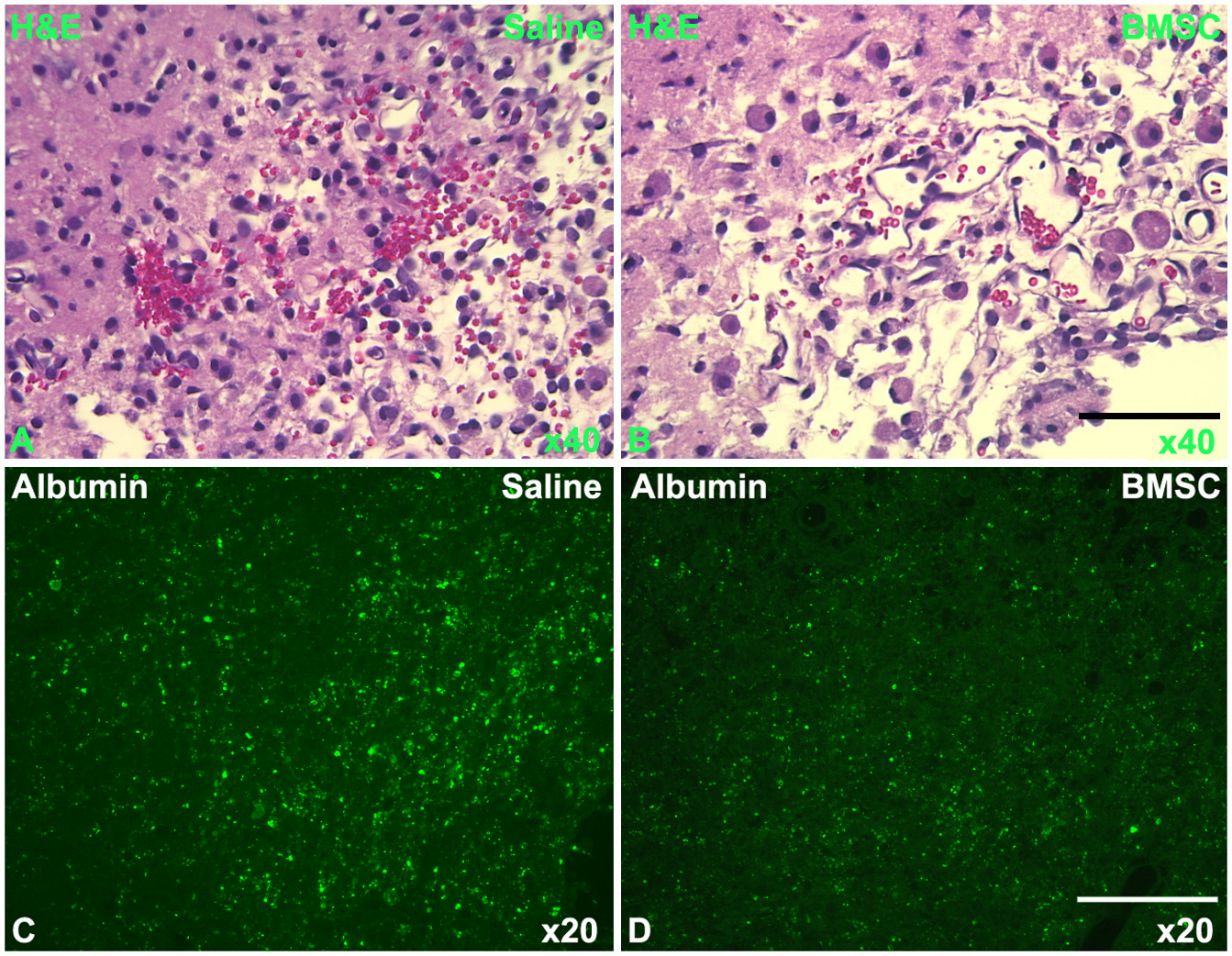
Histology of BBB disruption and hemorrhage. With the histological H&E stained sections under the light microscope, hemorrhage was found with more red cells and more diffusively distributed in the ischemic cerebral tissue in the control T2DM rat (A, 40x), than in the BMSC treated T2DM rat (B, 40x). Albumin stain demonstrated that the BBB leakage was more severe in the control T2DM rat (C, 20x) than in the BMSC treated T2DM rat (D, 20x). Bars: 50μm in B & 100μm in D.

Hemorrhage was histologically evaluated by counting erythrocytes outside of blood vessels in the cerebral parenchymal tissue. Under the light microscope, H&E stained coronal sections showed that red cells were diffusively present in the ischemic cerebral tissue in the control T2DM rat, as indicated in Fig 4A; while fewer red blood cells were present and were localized to a smaller region in the BMSC treated T2DM rat than in the control T2DM rat (Fig 4B). Measurements of red blood cells with H&E stain were 13.3±4.2 percent versus 7.1±3.8 percent for the control and BMSC treated rats, respectively, and no significant differences were found. Albumin stain provides a means to assess BBB leakage for large molecules. The albumin immunoreactive spots outside of blood vessels in the brain parenchymal tissue were larger and more dense for the control T2DM rat (Fig 4C) than that for the BMSC treated rat (Fig 4D). Measurements of percent albumin stain areas were 5.4±3.0 percent versus 2.0±0.9 percent for the control and BMSC treated rats, respectively, with a significant difference (p<0.05). Thus, coinciding with the MRI measurements, histological results of H&E and albumin stainings for BBB damage demonstrated that BMSC treatment of stroke in T2DM rats significantly reduced cerebral vascular leakage after stroke and substantially reduced hemorrhage compared to the control T2DM rats.

Neurological function graded by mNSS was performed. The BMSC treatment of stroke in T2DM rats significantly (p<0.05) improved neurological function of rats at 5 weeks after stroke with the mNSS of 6.33±1.50 for the saline treated T2DM rats and 5.11±0.60 for the BMSC treated rats, respectively.

## Discussion

BMSC treatment of stroke in previous experimental studies demonstrated differential therapeutic effects on vascular permeability in non-diabetic rats, where the treatment significantly reduced BBB leakage [26], and in T1DM rats, where the treatment increased BBB leakage [11]. Thus, there is a need to clarify the therapeutic effects of BMSC on vascular permeability in T2DM stroke rats. By employing CE-T1WI with gadopentetate, the present study demonstrates that, compared with the saline treatment, BMSC therapy administered at 3 days after stroke significantly (p<0.05) decreases BBB leakage in T2DM rats starting at 1 week after stroke, hence, leading to a significant (p<0.05) reduction of hemorrhagic spots starting from 3w after stroke and a marginally significant (p<0.08) reduction of hemorrhagic volume at 5w after stroke demarcated from SWI. The differences in vascular response of T1DM and T2DM stroke rats to BMSC treatment may be dependent on the time at which treatment is initiated, where cell administration were at 1 day and 3 days after stroke in T1DM and T2DM, respectively, and not necessarily on pathophysiological differences between T1DM and T2DM [11].

Delayed BMSC treatment of stroke in rats is not neuroprotective, and does not reduce infarction volume. It is neurorestorative. Neurorestorative approaches, essentially treat the intact tissue to promote neurovascular remodeling which enhances neurological recovery. Examples of neurovascular remodeling include angiogenesis [27], neurogenesis [18], oligodendrogenesis [28], synaptogenesis and axonal sprouting throughout the central nervous system [29, 30].

By employing dynamic T2WI measurements, we found that treatment of stroke with BMSC in T2DM rats does not reduce infarction volume during 5 weeks after stroke, compared to saline treated T2DM rats. Thus, administration of BMSCs initiated at 3 days after stroke in T2DM rats is not neuroprotective. However, the reduction of BBB leakage may be a biomarker of BMSC-induced neurorestorative effects. These data are consistent with prior reports that cell treatment administered at or more than 24 hours post stroke to non-diabetic rats or to T1DM rats does not reduce ischemic lesion volumes [11, 31]. Immunohistochemical measurements of infarction, BBB leakage and hemorrhage at 5w after stroke present consistent results with MRI measurements.

Hyperglycemia induces biochemical changes within endothelial cells [2], leading to vascular endothelial cell dysfunction, and increased vascular permeability [3]. As a result, more severe vascular damage has been consistently documented in stroke patients and animals with diabetes [3, 4]. In the present MRI study, gadopentetate, molecular weight of approximate 1KDa, was employed as a hallmark of BBB disruption. The intact BBB can hinder crossing of gadopentetate. However, because of breakdown of basal lamina with loss of astrocyte and endothelial cell contact, disruption of the BBB after ischemia allows accumulation of gadopentetate out of vessels in brain parenchyma, which primarily increases image intensity of cerebral tissue where it accumulates, leading to T1WI enhancement [20]. The enhancement may be interpreted as BBB damage leading to leakage of gadopentetate out of vessels into brain tissue, and not as increased vascular volume, since temporal functions of contrast concentration in cerebral vessels and tissue differ in response to the tail vein injection of the contrast, where the gadopentetate concentration in cerebral vessels rapidly rises and falls, while the gadopentetate concentration in cerebral tissue decreases very slowly after rising to its peak. Thus, BMSC treatment reduces gadopentetate enhanced volume after stroke in T2DM rats, compared with control rats, which implies that BMSC treatment decreased BBB disruption.

CE-T1WI with the administration of Gd-DTPA demonstrated different temporal features of blood vascular permeability between the control and BMSC treated T2DM rats, as indicated in Fig 2 from two representative animals. The contrast enhanced images, by subtraction of pre-contrast from post-contrast images of CE-T1WI, exhibited regions with elevated intensity in ischemic cerebral tissue, which indicated BBB damage leading to leakage of Gd-DTPA out of vessels into brain tissue.

With the same administration of gadopentetate, the differences between CE-T1WI and DCE-MRI are as follows: CE-T1WI is a convenient and quick method to detect BBB leakage in brain by comparing pre and post gadopentetate images, while the DCE-MRI method provides quantitative BBB permeability rate, K_i_, by modeling analysis of dynamic images with gadopentetate enhancement [23]. Measurements from CE-T1WI images and K_i_ maps are correlated in the ischemic brain of rats after stroke [20]. In the present study, as indicated in Fig 2, K_i_ maps and subtracted CE-T1WI images exhibit similar temporal patterns of BBB disruption in T2DM rats after stroke with or without the BMSC treatments. According to Eq 1, the Patlak plot is a straight line, and there is no information to estimate the interstitial volume. The ratio of permeability-surface product over flow is far less than 1, and the flow effect on K_i_ can be neglected. Permeability (K_i_) and vascular volume (V_p_) are separated in Eq 1. Hence, the K_i_ may be interpreted as specific for endothelial permeability [32].

**Fig 5:**
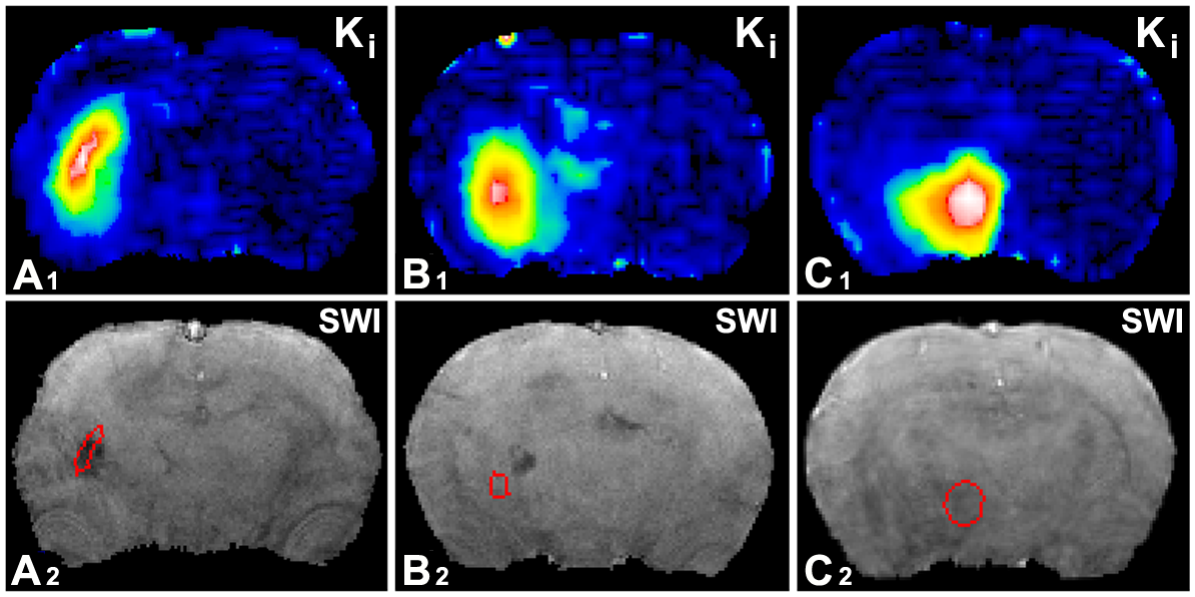
Mismatch of BBB disruption and hemorrhage. The locations of BBB disruption with high permeability K_i_ values of gadopentetate and the spots of hemorrhage with hypointensity of SWI, were found overlapped (A1 & A2), dislocated (B1 & B2), i.e., did not overlap, and inconsistent (C1 & C2), i.e., elevated permeability without detectable hemorrhage.

The evaluation of BBB disruption employing CE-T1WI depends on the size of contrast agent used in the experiment [20]. BBB damage increases permeability to small molecules at an early stage after stroke, such as gadopentetate. At later times, proteins or cells, e.g. albumin or erythrocytes, may penetrate the disrupted BBB. Erythrocytes die after leaving a ruptured blood vessel, and release the hemoglobin into the extracellular space. Extracellular hemoglobin loses oxygen and becomes paramagnetic deoxyhemoglobin, or can be engulfed by phagocytic cells and degraded into hemosiderin. SWI can detect changes of magnetic susceptibility induced by deoxyhemoglobin or hemosiderin. Thus, the CE-T1WI evaluation for BBB leakage need not be correlated with the SWI measurements for hemorrhage. As indicated in Fig 5, three pairs of images typically demonstrated different situations of BBB leakage and hemorrhage by CE-T1WI and SWI, respectively. Therefore, unlike the significant differences in volumes of BBB leakage detected by CE-T1WI, hemorrhagic volumes, detected by SWI, only exhibit marginally significant differences between the BMSC and saline treated T2DM rats after stroke.

HT after ischemia mostly occurs during the first week after stroke, and blood can persist in brain tissue and be continuously detected by SWI in six weeks after stroke in non-diabetic rats [24]. In contrast, stroke causes chronic and widespread BBB damage in T2DM rats [9]. Furthermore, according to our results, HT after stroke in T2DM rats, differs from that in the non-diabetic rats, results in multiple hemorrhagic spots in SWI (Fig 3), mostly, during the first week after stroke (Figs 1D and 3). Thus, treatment with BMSC at 3 days after onset of stroke in the present study essentially misses the first half week of hemorrhagic time window, and can only partly reduce HT by subsequently ameliorating damage of BBB. From this point of view, although hemorrhagic volume did not exhibit a significant difference between the BMSC and saline treated T2DM rats in 5w after stroke, BMSC treatment of stroke effectively protected BBB from further damage in T2DM rats, leading to a significant reduction of HT spots starting from 3 weeks after stroke (Fig 1D). However, in addition to decreasing hemorrhagic spots, BMSC treatment increases vascular endothelial growth factor expression and angiogenesis [27], which may lead to the marginally significant (p<0.08) reduction of hemorrhagic volume in treated T2DM rats at 5w after stroke, compared with the controls (Fig 1C).

In the present study, CE-T1WI detects BBB leakage of gadopentetate, and modulus images are reconstructed from SWI and employed to detect deoxyhemoglobin or hemosiderin as hemorrhagic evidence in the ischemic brain of T2DM rats. MRI markers differ from histological stains for either BBB leakage of albumin or hemorrhagic evidence of erythrocytes. Thus, the histological albumin and H&E measurements provide evidence to support MRI results, which demonstrated that BMSC treatment significantly reduced BBB disruption and leads to reduced hemorrhage after stroke in T2DM rats, compared with the vehicle treated T2DM rats.

In summary, the hypothesis of this study was successfully tested that the delayed, i.e. 3 days after stroke, treatment of T2DM rats with BMSC promotes neurorestorative effects, reduces BBB disruption, and improves functional outcome. These gadopentetate related MRI findings are consistent with, and supported by histological results of albumin and H&E stainings for BBB disruption.

## Abbreviations

BBB: blood-brain barrier
BMSC: bone marrow stromal cells
CE-T1WI: contrast enhanced T1-weighted imaging
DCE-MRI: dynamic contrast-enhanced
DGE: dual gradient echo
Gadopentetate: gadolinium-diethylenetriamine penta-acetic acid
HFD: high fat diet
H&E: hematoxylin and eosin
HT: hemorrhagic transformation
K_i_: the estimated blood-to-brain transfer rates of contrast agent
MCA: middle cerebral artery
mNSS: modified neurological severity scores
MRI: magnetic resonance imaging
PBS: phosphate-buffered saline
STZ: streptozotocin
SWI: susceptibility weighted imaging
T1DM: type 1 diabetes mellitus
T1WI: T_1_-weighted imaging
T2DM: type 2 diabetes mellitus
T2WI: T_2_-weighted imaging
TE: time of echo
TR: time of repetition
